# Supplementary material for: MetaRibo-Seq measures translation in microbiomes
Source: Nat Commun. 2020 Jun 29;11:3268. doi: 10.1038/s41467-020-17081-z (PMC7324362; doi:10.1038/s41467-020-17081-z)
Supplement: Supplementary file 10 — Supplementary Data 7 [file 41467_2020_17081_MOESM10_ESM.zip › File2/Confidence_VeryHigh_Taxonomy/298792_out.krona.html]

Javascript must be enabled to view this page.

members
magnitude
magnitudeUnassigned
count
unassigned
taxon
rank

298792\_out

6

superkingdom
2
6

phylum
1239
6

2292890

SRS104197\_contig\_number\_contig-100\_17.6176
1
species

class
186801
5

186802
5
order

186803
5
family

1506553
2
genus

84030

SRS075398\_contig\_number\_contig-100\_4745.4746
1
species


SRS063127\_contig\_number\_contig-100\_2635.2636
1531
1
species


SRS018739\_contig\_number\_14192
1898203
1
species

genus
1164882
1

1

SRS054687\_contig\_number\_contig-100\_19782.42919
467210
species

1
397288

SRS052078\_contig\_number\_contig-100\_1749.29409
species
